# Root Growth and Enzymes Related to the Lignification of Maize Seedlings Exposed to the Allelochemical L-DOPA

**DOI:** 10.1155/2013/134237

**Published:** 2013-11-14

**Authors:** Rita de Cássia Siqueira-Soares, Anderson Ricardo Soares, Angela Valderrama Parizotto, Maria de Lourdes Lucio Ferrarese, Osvaldo Ferrarese-Filho

**Affiliations:** Laboratory of Plant Biochemistry, Department of Biochemistry, University of Maringá, Av. Colombo 5790, 87020-900 Maringá, PR, Brazil

## Abstract

L-3,4-Dihydroxyphenylalanine (L-DOPA) is a known allelochemical exuded from the roots of velvet bean (*Mucuna pruriens* L. Fabaceae). In the current work, we analyzed the effects of L-DOPA on the growth, the activities of phenylalanine ammonia-lyase (PAL), tyrosine ammonia-lyase (TAL), and peroxidase (POD), and the contents of phenylalanine, tyrosine, and lignin in maize (*Zea mays*) roots. Three-day-old seedlings were cultivated in nutrient solution with or without 0.1 to 2.0 mM L-DOPA in a growth chamber (25°C, light/dark photoperiod of 12/12, and photon flux density of 280 **μ**mol m^−2^ s^−1^) for 24 h. The results revealed that the growth (length and weight) of the roots, the PAL, TAL, and soluble and cell wall-bound POD activities decreased, while phenylalanine, tyrosine, and lignin contents increased after L-DOPA exposure. Together, these findings showed the susceptibility of maize to L-DOPA. In brief, these results suggest that the inhibition of PAL and TAL can accumulate phenylalanine and tyrosine, which contribute to enhanced lignin deposition in the cell wall followed by a reduction of maize root growth.

## 1. Introduction

Allelochemicals are found in many higher plants. These compounds can be regularly released into the environment by various mechanisms, such as leaching by rainwater, excretion or exudation from roots, and by the natural decay of parts of plants lying above or below the ground [1]. In contact with the rhizosphere or the bulk soil, allelochemicals may be absorbed by receptor plants and exert their action. Although allelochemicals play a relevant role in chemical plant-plant interactions, the primary mode of action has not been established for any of them. In general, they suppress seed germination, cause injury to root growth, and inhibit seedling growth. Moreover, they alter several physiological processes such as water and mineral uptake, foliar expansion, photosynthesis, membrane permeability, cell morphology, protein synthesis, cell respiration, and enzyme activity [2, 3].

Velvet bean (*Mucuna pruriens* L. Fabaceae) is an important plant species in tropical countries like India and other parts of Central and South America [4]. It has long been cultivated in tropical regions for intercropping with maize, sorghum, and millet and for providing benefits such as symbiotic nitrogen fixation, weed smothering, nutrient recycling, suppression of the nematode population, control of erosion, and soil improvement [5, 6]. Many secondary compounds are produced by the seeds, leaves, and roots of velvet bean, but the main compound is the nonprotein amino acid L-3,4-dihydroxyphenylalanine (L-DOPA). This allelochemical constitutes 0.5–1.5% of the fresh leaves weight [7] and 6–9% of the dry seed weight [8]. The yield of fresh weight leaves and stems from plants ranges from 20 to 30 ton ha^−1^, and, as a consequence, velvet bean can add 100–450 kg ha^−1^ of L-DOPA to soils [9, 10]. The concentration of L-DOPA released from velvet bean roots may reach 1 ppm in water culture solution and 50 ppm in the vicinity of roots [11], both of which are sufficient to affect the growth of neighboring plants by reducing seed germination and suppressing root growth [7, 10, 12–15]. However, the mechanism of action of L-DOPA is not well clarified in plants.

L-DOPA is a precursor of many alkaloids, catecholamines, flavonoids, melanin, and phenylpropanoids in plants [16]. The phenylpropanoid pathway is one of the most important metabolic routes because it synthesizes phenolic compounds and many other secondary chemical agents, including lignin. Some enzymes of this metabolic pathway, such as phenylalanine ammonia-lyase (PAL), tyrosine ammonia-lyase (TAL), and peroxidase (POD), are associated with the synthesis and polymerization of monolignols and, consequently, with premature lignification [17, 18]. We have demonstrated previously that soybean (*Glycine max*) seedlings had impaired root growth and increased lignin production after L-DOPA treatment [19]. However, no reports on the effects of exogenous L-DOPA on maize roots are available to date. In view of this open question, the purpose of work was to evaluate the effects of L-DOPA on maize root growth and the activities of some enzymes related to the phenylpropanoid pathway, such as PAL, TAL, and POD. The phenylalanine, tyrosine, and lignin levels also were evaluated.

## 2. Materials and Methods

### 2.1. General Procedures

Maize (*Zea mays* cv. IPR-114) seeds were surface-sterilized with 2% sodium hypochlorite for 5 min, rinsed extensively with deionized water, and dark-germinated at 25°C on two sheets of moistened filter paper. Twenty-five 3-day-old seedlings of uniform size were supported by an adjustable acrylic plate and dipped into a 10 × 16 cm glass container filled with 200 mL of half-strength Hoagland's solution (pH 6.0), with or without 0.1 to 2.0 mM L-DOPA. All nutrient solutions were buffered with 67 mM potassium phosphate buffer to eliminate the effects of very low pH. The containers were kept in a growth chamber for 24 h at 25°C, with a light/dark photoperiod of 12/12 h and a photon flux density of 280 **μ**mol m^−2^ s^−1^. The roots were measured before incubation and at the end of the experiment, and the difference in length was calculated for all of the samples. The fresh root weight was determined immediately after incubation, and the dry weight was estimated after oven-drying at 80°C until a constant weight was achieved. L-DOPA was purchased from Sigma-Aldrich (St. Louis, MO, USA), and all other reagents used were of the purest grade available or of chromatographic grade.

### 2.2. Enzymatic Assays

PAL was extracted as described by Ferrarese et al. [20]. Fresh roots (2 g) were ground at 4°C in 0.1 M sodium borate buffer (pH 8.8); the homogenate was centrifuged (2,200 ×g, 15 min) and the supernatant was used as the enzyme preparation. The reaction mixture contained 100 **μ**mol sodium borate buffer, pH 8.7, and a suitable amount of enzyme extract in a final volume of 1.5 mL. The mixture was incubated at 40°C for 5 min. To initiate the reaction, 15 **μ**mol of L-phenylalanine was added, and the reaction was stopped after 1 h by the addition of 50 **μ**L of 5 M HCl. The sample was filtered through a 0.45 **μ**m disposable syringe filter and analyzed (20 **μ**L) in a high performance liquid chromatography (HPLC) system (Shimadzu, Tokyo, Japan). A reverse-phase Shimpack CLC-ODS column (150 mm × 4.6 mm, 5 *μ*m) was used at 30°C with an equivalent precolumn. The mobile phase was 70% methanol (v/v), with a flow rate of 0.5 mL min^−1^ for an isocratic run of 10 min. UV detection was performed at 275 nm. The product of PAL, *t*-cinnamate, was identified by comparing its retention time with those of standard compounds. PAL activity is expressed as *μ*mol *t*-cinnamate h^−1^ g^−1^ fresh weight.

TAL was extracted as described by Khan et al. [21]. Fresh roots (1 g) were ground at 4°C in 2.5 mL of 50 mM Tris-HCl 0.1 M buffer (pH 8.5). The homogenate was centrifuged (2,200 ×g, 10 min) and the supernatant was used as the enzyme preparation. The reaction mixture (100 **μ**mol Tris-HCl buffer, pH 8.5, and a suitable amount of enzyme extract in a final volume of 0.95 mL) was incubated at 40°C for 5 min. To initiate the reaction, 5.5 **μ**mol of L-tyrosine was added, and the reaction was stopped after 1 h by the addition of 50 **μ**L of 5 M HCl. The sample was filtered through a 0.45 **μ**m disposable syringe filter and analyzed (20 **μ**L) by HPLC as described earlier. The mobile phase was methanol : acetic acid 4% (30% : 70%), with a flow rate of 0.8 mL min^−1^ for an isocratic run of 10 min. UV detection was carried out at 320 nm. *p*-Coumarate, the product of TAL reaction, was identified by comparing its retention time with standard compounds. Parallel controls without L-tyrosine or with *p*-coumarate (added as an internal standard in the reaction mixture) were performed. TAL activity is expressed as *μ*mol *p*-coumarate h^−1^ g^−1^ fresh weight.

For the POD assay, fresh roots (0.5 g) were ground in a mortar with 0.01 g of PVP and 5 mL of 67 mM phosphate buffer (pH 7.0). The extract was centrifuged (2,200 ×g, 5 min), and the supernatant was used to determine the activity of soluble POD. The pellet was incubated in 1 M NaCl (2 mL for 1 h at 4°C). The homogenate was centrifuged (2,200 ×g, 5 min), and the supernatant contained the cell wall (ionically) bound POD. The enzyme activities were determined according to methods described by dos Santos et al. [22]. The reaction mixture (3 mL) contained 25 mM sodium phosphate buffer (pH 6.8), 2.58 mM guaiacol, and 10 mM H_2_O_2_. The reaction was initiated by the addition of the enzyme extract. The oxidation of guaiacol was followed for 5 min at 470 nm, and the enzyme activity was calculated from the extinction coefficient (25.5 mM^−1^ cm^−1^). POD activities are expressed as *μ*mol tetraguaiacoquinone min^−1^ g^−1^ fresh weight.

### 2.3. Phenylalanine and Tyrosine Quantification

Samples (5.0 mg of dry root) that had been previously defatted were transferred to Pyrex ampoules (10 × 150 mm) that had been pyrolyzed at 400°C for 8 h. Next, 0.5 mL of aqueous 6 M HCl, which had been doubly distilled at 104°C and contained 0.1% phenol, was added to each sample. Vials were sealed under vacuum and placed in an oven at 110°C for 22 h. After acid hydrolysis, the solution was dried using rotary evaporation and resuspended in 0.17 M sodium citrate buffer (pH 2.2) containing 15% polyethylene glycol (PEG 400) and 0.4% thiodiglycol. Samples (0.9 mL) were loaded into a cation exchange column (Resin: PC 6A amino acid analysis resin pierce) and eluted by pH and ionic strength (short column pH 5.28, long column pH 3.25, and additional pH 4.25). After chromatographic separation, the amino acids eluted from the column were reacted with ninhydrin in a boiling water bath (100°C for 15 min), and the products of the reaction were detected colorimetrically at 570 nm [23]. The phenylalanine and tyrosine contents are expressed as mg g^−1^ dry weight.

### 2.4. Lignin Quantification

After the incubation period, dry roots (0.3 g) were homogenized in 50 mM potassium phosphate buffer (7 mL, pH 7.0) with a mortar and pestle and transferred into a centrifuge tube [24]. The pellet was centrifuged (1,400 ×g, 4 min) and washed by successive stirring and centrifugation as follows: 2x with phosphate buffer pH 7.0 (7 mL); 3x with 1% (v/v) Triton X-100 in pH 7.0 buffer (7 mL); 2x with 1 M NaCl in pH 7.0 buffer (7 mL); 2x with distilled water (7 mL); and 2x with acetone (5 mL). The pellet was dried in an oven (60°C, 24 h) and cooled in a vacuum desiccator. The dry matter obtained was defined as a protein-free cell wall fraction. Lignin content was determined by the acetyl bromide method [25]. Sample (20 mg) of protein-free cell wall fraction obtained earlier was placed into a screw-cap centrifuge tube containing 0.5 mL of 25% acetyl bromide (v/v in glacial acetic acid) and incubated at 70°C for 30 min. After complete digestion, the samples were quickly cooled on ice and mixed with 0.9 mL of 2 M NaOH, 0.1 mL of 7.5 M hydroxylamine-HCl, and 2 mL of glacial acetic acid. After centrifugation (1,400 ×g, 5 min), the absorbance of the supernatant was measured at 280 nm. A standard curve with lignin (alkali, 2-hydroxy-propyl ether, Aldrich 37,096-7) was generated, and the absorptivity (*ε*) value obtained was 16.4 g^−1^ L cm^−1^. The results are expressed as mg lignin g^−1^ cell wall.

### 2.5. Statistical Design

The experimental design was completely randomized, with each point on the plot representing one glass container of 25 seedlings. The data are expressed as the means of three to six independent experiments ± SE. Significant differences were verified by one-way analysis of variance (ANOVA) using the *GraphPad Prism* package (Version 2.0, GraphPad Software Inc., USA, 1995). Differences between parameters were evaluated by the Dunnett's multiple comparison test, and *P* values < 0.05 were adopted as the minimum criterion for statistical significance. 

## 3. Results

In comparison with the control, the root lengths were reduced by 26.9% and 52.9% for 1.0 and 2.0 mM treatments, respectively (Table 1). The effects of the allelochemical were also evident for root weights, which significantly decreased by 11.1% and 25.6% (fresh weight) and 9.5% and 16.3% (dry weight) after exposure to 1.0 and 2.0 mM when compared with the respective controls.

**Figure 1:**
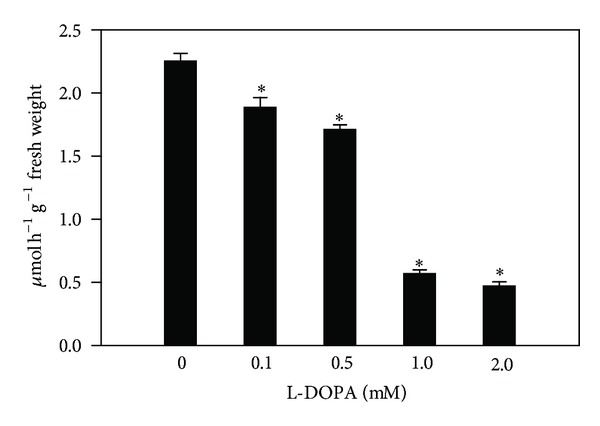
Effects of L-DOPA on phenylalanine ammonia-lyase (PAL). *Values (*N* = 3 ± SE) differ statistically (Dunnett's multiple comparison test) from control (*P* < 0.05).

**Figure 2:**
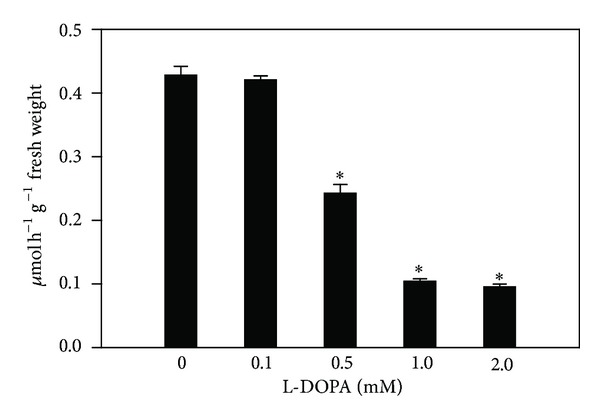
Effects of L-DOPA on tyrosine ammonia-lyase (TAL). *Values (*N* = 3 ± SE) differ statistically (Dunnett's multiple comparison test) from control (*P* < 0.05).

**Figure 3:**
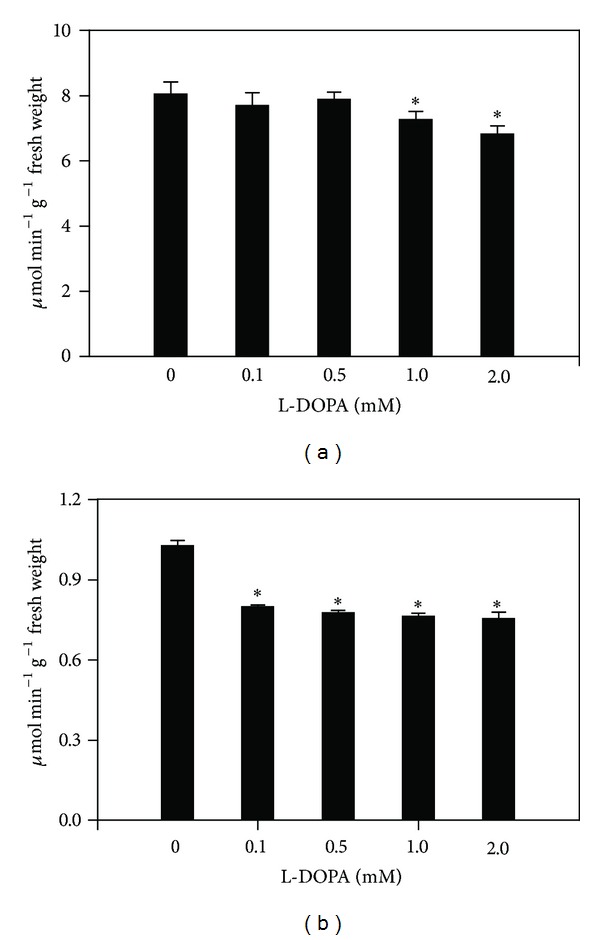
Effects of L-DOPA on soluble (a) and cell wall-bound (b) peroxidases (POD). *Values (*N* = 5 ± SE) differ statistically (Dunnett's multiple comparison test) from control (*P* < 0.05).

In agreement with the effects observed on root growth, the enzyme activity of seedlings treated with L-DOPA was also significantly different from those of controls. Roots exposed to L-DOPA significantly decreased PAL activities by 16.2% to 79% after treatment with 0.1 to 2.0 mM, respectively, in comparison with the control (2.25 ± 0.06 **μ**mol h^−1^ g^−1^ fresh weight) (Figure 1). The allelochemical also decreased TAL activities from 43.3 to 77.6% after 0.5 to 2.0 mM treatments, compared with the control (0.43 ± 0.01 **μ**mol h^−1^ g^−1^ fresh weight) (Figure 2). The soluble POD activities were decreased by 9.8 and 15.2% for 1.0 and 2.0 mM treatments, in comparison to the control (8.05 ± 0.38 **μ**mol h^−1^ g^−1^ fresh weight) (Figure 3(a)). On the other hand, the cell wall-bound POD activities were decreased after all L-DOPA treatments, that is, from 22.2 to 26.5%, when compared to the control (1.03 ± 0.03 **μ**mol h^−1^ g^−1^ fresh weight) (Figure 3(b)).

**Figure 4:**
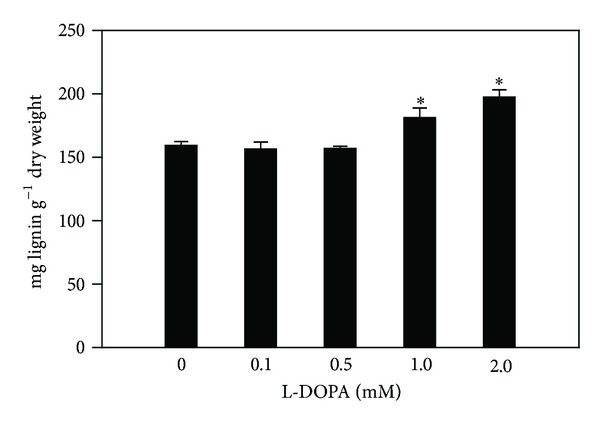
Effects of L-DOPA on lignin contents. *Values (*N* = 4 ± SE) differ statistically (Dunnett's multiple comparison test) from control (*P* < 0.05).

The lignin content increased from 15.3 to 25.7% after treatment with the two highest concentrations of L-DOPA, respectively, in comparison to the control (159.4 ± 3.01 mg g^−1^ dry weight) (Figure 4). The results obtained after exposure of maize seedlings to L-DOPA revealed that the content of phenylalanine and tyrosine increased by 46% and 18.9%, respectively, in comparison to the control (Table 2).

## 4. Discussion

In the current work, we showed that the growth (length and weight) of maize roots was significantly affected by L-DOPA (Table 1), which is a feature common to the effects of different allelochemicals in plants [2, 3]. The reduction of root growth by the action of L-DOPA at different concentrations has been described in several plant species [26–28] although some of them were resistant to the allelochemical [13]. Since L-DOPA reduced the growth of maize roots (Table 1), its role as a potent allelochemical has been strengthened.

Decrease in root length has been related to the cell wall lignification induced by allelochemicals. In general, lignification stiffens the cell wall with concomitant increases in PAL and POD activities. In fact, increases of PAL activity correlate with reduction of root growth and lignin production in maize, cucumber, and soybean exposed to the action of phenylpropanoid allelochemicals [22, 29–31]. In addition, soluble and cell wall-bound POD activities increase concomitantly with lignin production in roots of maize [29], cucumber [30], and soybean [22, 32, 33]. With respect to L-DOPA, Soares et al. [19] observed a reduced root length of soybean followed by increases in PAL and POD activities and lignin content.

In contrast to the abovementioned reports, we have found that L-DOPA decreased the maize root growth and PAL, TAL, and soluble and cell wall-bound POD activities (Figures 1 to 3) but slightly increased the lignin content at high concentrations (Figure 4). It is not a bottleneck effect, however, because the apparent contradiction can be refuted by the complexity of the shikimate and phenylpropanoid pathways in plants. The shikimate pathway, which leads to the synthesis of aromatic amino acids such as phenylalanine and tyrosine, and the phenylpropanoid pathway towards the synthesis of lignin are clearly interconnected [18]. It is well known that lignin consists of *p*-hydroxyphenyl (H), guaiacyl (G), and syringyl (S) monomers, which can vary between plant species and different tissues and localization of the cell wall [17, 34, 35]. In addition to these main units, unconventional monomers can be formed from *p*-coumarates, acetates, acylated hydroxycinnamyl alcohols, *p*-hydroxybenzoates, dihydrohydroxycinnamyl alcohols, and tyramine ferulate [18, 36, 37]. Furthermore, Ralph et al. [38] suggested that the plants simply require a polymer with specific mechanical properties, and that the usual composition of lignin is not important. Due to these findings, it is not surprising that lignin can be maintained at a relatively constant level, even when enzymes involved in the synthesis of its precursors (PAL and TAL) and polymerization (POD) are suppressed or compromised. Such evidences can explain the slight increase of lignin content observed herein (Figure 4).

It has become clear from this study that L-DOPA increased the levels of phenylalanine and tyrosine in maize roots (Table 2). Some studies suggest that L-DOPA can be metabolized in these two amino acids. For example, Nakajima et al. [12] observed a significant accumulation of L-DOPA and free amino acids in the roots of cucumber in comparison to untreated plants. Also, enhanced levels of phenylalanine and tyrosine have been found in *Echinochloa crus-galli*, lettuce [26] and soybean [15] after L-DOPA treatments. According to the latter report, the accumulation of both amino acids can be a result of the detoxification of L-DOPA. Here, we believe that the inhibition of PAL and TAL (Figures 1 and 2) can also contribute to the accumulation of phenylalanine and tyrosine, followed by incorporation of these amino acids into the cell wall. Evidences support this hypothesis. Carpita [39] reported that sections excised from maize coleoptiles incorporated radioactivity from phenylalanine (40%) and tyrosine (30%) into structural components of the cell wall. Moreover, linkages of phenolic side-chains of tyrosine residues are directly involved in lignin deposition into the cell wall [40]. In addition, a tyrosine-rich cell wall protein has been specifically located in the secondary cell wall and can be involved in the lignin deposition [41]. Finally, McDougall et al. [42] observed that the presence of tyrosine residues increased the incorporation of monolignol coniferyl alcohol into lignin-like polymers.

## 5. Conclusion

If our hypothesis is correct, L-DOPA can be metabolized to phenylalanine and tyrosine in maize roots or, itself, can inhibit enzymes of the phenylpropanoid pathway, such as PAL and TAL, accumulating amino acids. A possible incorporation of phenylalanine and tyrosine into the cell wall can increase the presence of unconventional monomers followed by lignin deposition. As a consequence, the cell expansion is restricted and the root growth is reduced. To give a clear answer to this hypothesis, additional efforts are required to evaluate the structural characterization of the supposed unconventional lignin, including a more specific analysis of phenylalanine and tyrosine residues into the cell wall after L-DOPA treatments. This is the challenge faced by a new study that is currently in progress.

## Figures and Tables

**Table 1:** Changes in root length, root fresh and dry weights of maize seedlings treated with L-DOPA for 24 h.

L-DOPA (mM)	Root length (cm)	%	Fresh weight (g)	%	Dry weight (g)	%
0	3.38 ± 0.032		2.15 ± 0.052		0.148 ± 0.001	
0.1	3.25 ± 0.187^ns^		2.06 ± 0.147^ns^		0.145 ± 0.004^ns^	
0.5	2.98 ± 0.140^ns^		1.96 ± 0.020^ns^		0.138 ± 0.002^ns^	
1.0	2.47 ± 0.069*	26.9	1.89 ± 0.083*	11.1	0.134 ± 0.003*	9.5
2.0	1.59 ± 0.061*	52.9	1.68 ± 0.044*	25.6	0.124 ± 0.004*	16.32

Means (*N* = 5 ± SE) significantly smaller than the experiment control (Dunnett's multiple comparison test) are marked*. ns: not significant at 0.05 level. The symbol % represents inhibition of statistically significant means when compared to control (0 mM).

**Table 2:** Changes in the levels of tyrosine and phenylalanine of maize seedlings treated with L-DOPA for 24 h.

L-DOPA (mM)	Tyrosine (*μ*mol g^−1^ fresh weight)	Phenylalanine (*μ*mol g^−1 ^fresh weight)
0	7.9 ± 0.152	2.18 ± 0.033
0.5	9.4 ± 0.115*	3.18 ± 0.174*

Means (*N* = 3 ± SE) significantly smaller than the experiment control (Student's *t* test) are marked*.
